# Internet Memes as Drivers of Health Narratives and Infodemics: Integrative Review

**DOI:** 10.2196/77029

**Published:** 2025-12-19

**Authors:** Alvaro Carmona Pestaña, Iván Herrera-Peco, Beatriz Jiménez-Gómez, Carolina Suárez-Llevat

**Affiliations:** 1 Departamento de Ciencias de la Salud y Biomedicina, Facultad de Ciencias de la Salud, Universidad Loyola Andalucía, Avda de las Universidades s/n, Dos Hermanas, 41704, Spain, 34 624295159; 2 Socialhealthcare-UAX Research Group, Facultad de Ciencias Biomédicas y de la Salud, Universidad Alfonso X el Sabio, Villanueva de la Cañada, Spain; 3 Facultad de Ciencias de la Salud-HM, Universidad Camilo José Cela, Villanueva de la Cañada, Madrid, Spain; 4 Instituto de Investigaciones Sanitarias HM Hospitales, Madrid, Madrid, Spain; 5 Department of Nursing, Faculty of Medicine, Health and Sports, Universidad Europea, Madrid, Spain; 6 Departamento de Psicología, Facultad de Ciencias Biomédicas y Deportes, Universidad Europea de Andalucía, Málaga, Spain

**Keywords:** health communication, social media, internet memes, infodemic, infodemiology, misinformation, public health campaigns

## Abstract

**Background:**

Digital media memes have emerged as influential tools in health communication, particularly during the COVID-19 pandemic. While they offer opportunities for emotional engagement and community resilience, they also act as vectors for health misinformation, contributing to the global infodemic. Despite growing interest in their communicative power, the role of memes in shaping public perception and misinformation diffusion remains underexplored in infodemiology.

**Objective:**

This integrative review aims to analyze how memes influence emotional, behavioral, and ideological responses to health crises, and to examine their dual role as both contributors to and potential mitigators of infodemics. The paper also explores strategies for integrating memes into public health campaigns and infodemic management.

**Methods:**

A comprehensive literature search was conducted across 3 major databases (MEDLINE, Scopus, and Web of Science), identifying a total of 386 records. Following duplicate removal and eligibility screening, 14 peer-reviewed studies published between 2020 and 2025 were included. An integrative narrative approach was used to synthesize evidence on social media behavior, misinformation dynamics, and digital health campaigns. The analysis was grounded in infodemiological and infoveillance frameworks as established by Eysenbach, incorporating insights from psychology, media studies, and public health.

**Results:**

Memes function as emotionally salient and visually potent carriers of health-related narratives. While they can simplify complex messages and foster adaptive humor during crises, they are also susceptible to distortion, particularly in echo chambers and conspiracy communities. Findings reveal that misinformation-laden memes often leverage humor and disgust to bypass critical thinking, and their viral potential is linked to emotional intensity. However, memes have also been successfully integrated into prebunking strategies, increasing engagement and reducing susceptibility to false claims when culturally tailored. The review identifies key mechanisms that enhance or hinder the infodemiological value of memes, including political orientation, digital literacy, and narrative framing.

**Conclusions:**

Memes are a double-edged sword in the context of infodemics. Their integration into infodemic surveillance and digital health campaigns requires a nuanced understanding of their emotional, cultural, and epistemic effects. Public health institutions should incorporate meme analysis into real-time infoveillance systems, apply evidence-based meme formats in prebunking efforts, and foster digital literacy that enables critical meme consumption. Future infodemiology research should further explore the long-term behavioral impacts of memetic misinformation and the scalability of meme-based interventions.

## Introduction

### Background

The term “meme” was first conceptualized by Dawkins in 1976 [1] in *The Selfish Gene* as a unit of cultural transmission analogous to genes in biological evolution. Memes, in this sense, are replicable units of information that spread through imitation, adapting and evolving as they traverse social networks.

The integration of digital media into public health discourse has reshaped the way scientific information is communicated to the public. During the COVID-19 pandemic, for example, memes played a dual role: amplifying preventive messages in some cases, while spreading pseudoscientific claims and distrust in others. As noted by the World Health Organization, the COVID-19 crisis has been accompanied by an “infodemic”—an overabundance of information, including deliberate attempts to disseminate misinformation and disinformation, which poses a serious threat to public health communication efforts [2]. Through this period, public health influencers (PHIs) emerged as pivotal figures in bridging the gap between formal medical expertise and public engagement. MacDonald and Wiens [3] argue that these PHIs leveraged memetic bricolage techniques—such as stop motion, collage, infographics, and placarding—to distil complex health information into digestible, shareable content across platforms such as Instagram, TikTok, and Twitter. Their study highlights how this form of science communication not only combated misinformation but also worked toward equitable health advocacy, making public health messaging more accessible to diverse audiences. The role of PHIs in challenging health inequities is particularly notable. Their efforts illustrate that digital advocacy can pressure governmental institutions to enact more transparent, community-driven health policies. By leveraging social media affordances, PHIs were able to redirect the spread of disinformation back toward evidence-based facts, reinforcing the notion that strategic digital engagement is crucial for modern public health interventions [4].

Moreover, MacDonald and Wiens [3] argue that internet memes extend beyond their entertainment value, functioning as strategic communicative tools capable of fostering meaningful engagement with nonexpert audiences on complex topics related to health and science. This perspective is further substantiated by Occa et al [5], who, through a systematic review of online health-related memes, identified these visual-textual artifacts as a promising message strategy for health promotion and education. Their findings indicate that while memes are a promising strategy for health education and awareness, their impact varies significantly based on their content, audience, and context. The review identified that most studies on health memes have focused on the COVID-19 pandemic, with other areas such as mental health, vaccination campaigns, and chronic disease awareness remaining relatively underexplored. Moreover, the review found that only a small fraction of studies has examined the direct impact of memes on behavioral health outcomes, underscoring the need for more experimental and longitudinal studies to assess the efficacy of meme-based interventions. Interestingly, this highlights that memes can serve not only as conduits for health communication but also as mechanisms for reducing stigma associated with certain medical conditions. By integrating humor and relatability, memes may facilitate difficult conversations around health issues, making them particularly useful in areas where traditional communication strategies struggle to engage audiences.

Reynolds and Boyd [6] conducted an exploratory descriptive analysis examining health care workers’ perspectives on the use of memes as an implementation strategy in infection prevention. Their findings suggest that memes can serve as effective tools for disseminating evidence-based practices, enhancing knowledge, and improving compliance among health care professionals. This aligns with the concept of memes functioning as “lively data”—dynamic, context-responsive units of digital discourse that adapt to evolving public concerns and sociocultural narratives [6].

The widespread nature of social media ensures that memes reach diverse demographics, but this also raises concerns regarding equity in health communication. In addition, policymakers and public health agencies must consider integrating digital literacy initiatives to equip individuals with the skills needed to critically evaluate meme-based health information before accepting or sharing it [7]. The landscape of health communication has undergone a significant transformation with the advent of digital media. Traditional health campaigns, which historically relied on television, radio, and print materials, are increasingly supplemented or even replaced by digital strategies that leverage the reach and engagement potential of social media platforms. Their effectiveness lies in their ability to engage users emotionally while simplifying complex medical concepts, making them particularly useful in environments where misinformation is prevalent [8].

Virality—the rapid dissemination of content through online social networks—is a key driver of meme efficacy in health communication. According to this, content that is emotionally evocative, novel, and has high informational use is more likely to be shared [9]. In the context of health-related content, positive sentiment and practical use enhance a paper’s likelihood of being shared via email or social media, contributing to broader public engagement. This shift has given rise to new modalities of public health messaging, among which memes—highly shareable and culturally relevant digital artifacts—have emerged as a potent tool for engagement, advocacy, and health education. Recent empirical studies highlight the role of memes in mobilizing the medical community for training and advocacy. Wang and colleagues [10] investigated the *GetWaivered* campaign, which used humor-based memes to encourage clinicians to register for opioid treatment training. Their study found that meme-driven content increased web traffic and enrollment in a multidimensional digital awareness campaign to increase Drug Enforcement Administration (DEA)-X waiver training courses, demonstrating the practical application of memes in professional medical education [10].

This duality positions memes at the heart of the current infodemic—a term defined by the World Health Organization as the overabundance of information, both accurate and false, that makes it difficult for individuals to find reliable guidance during a health crisis. Memes contribute to the dynamics of infodemics by shaping how risk is framed, which sources are trusted, and what behaviors are normalized or rejected [11].

To address these dynamics, this review adopts an infodemiological perspective, as proposed by Eysenbach [12,13], which seeks to understand the distribution, determinants, and impact of health information within digital environments. As a subfield of public health informatics, infodemiology integrates insights from epidemiology, media studies, and behavioral science to analyze how information—accurate or not—spreads through populations and influences health outcomes. Within this framework, memes are conceptualized not only as digital artifacts but also as epidemiological signals that warrant systematic observation.

This review also draws on the concept of infoveillance [14], or information surveillance, to highlight how viral content—particularly memetic formats—can serve as early indicators of emerging health sentiments and misinformation trends. By monitoring these content flows, public health actors may be able to anticipate narrative shifts, identify at-risk populations, and design targeted interventions.

In this context, this paper offers a narrative synthesis of recent empirical studies examining the communicative, cultural, and psychological dimensions of internet memes in health discourse. It aims to map both the risks and opportunities that memes pose in the digital health communication landscape and to offer infodemiologically grounded recommendations for researchers, communicators, and policymakers navigating the intersection of humor, misinformation, and public trust.

### Objectives

The primary aim of this narrative review is to critically analyze the impact of internet memes and viral content within the context of health communication, synthesizing recent empirical evidence to elucidate both the opportunities and challenges presented by these digital tools in public health promotion.

Specifically, this review seeks to evaluate (1) the role of memes and viral content in disseminating accurate, evidence-based medical information, including their effects on shaping public perceptions of health topics; (2) the effectiveness of memes in engaging diverse and traditionally hard-to-reach audiences, thereby enhancing participation and interaction in health communication efforts; (3) the potential of meme-based communication to mobilize health care professionals, examining how such digital strategies may influence clinical practices, professional education, and engagement in health advocacy campaigns; and (4) the challenges associated with misinformation propagated through memes and viral content, assessing how these formats may simultaneously contribute to and counteract health misinformation, misconceptions, and conspiracy beliefs.

## Methods

### Study Design

This narrative review was developed following a structured yet flexible approach, suitable for interpretative and thematic synthesis. While it does not follow the formal protocol of a systematic review, methodological rigor and transparency were prioritized throughout the process. To ensure clarity in the study selection process, a PRISMA (Preferred Reporting Items for Systematic Reviews and Meta-Analyses) flow diagram was constructed following the updated 2020 guidelines [15].

### Search Strategy

A comprehensive search of the literature was conducted in March 2025 across 3 major databases: MEDLINE, Scopus, and Web of Science. The search strategy combined MeSH (Medical Subject Headings) and free-text terms connected by Boolean operators. Terms such as *memes*, *internet meme*, *viral content*, *health communication*, *health promotion*, and *misinformation* were systematically combined to retrieve relevant literature across all 3 platforms. Search equations were adapted to each database’s syntax to ensure specificity and completeness.

The search queries were tailored to each database, using combinations of MeSH and relevant keywords, connected by Boolean operators (Table 1).

**Table 1.** Search equations of databases.

Database	Search equation
MEDLINE	("Memes"[ All Fields] OR "Internet Meme"[All Fields] OR "Social Media"[MeSH Terms]) AND ("Health Communication"[MeSH Terms] OR "Health Promotion"[MeSH Terms] OR "Misinformation"[MeSH Terms])
Scopus	TITLE-ABS-KEY(("memes" OR "internet meme" OR "viral content") AND ("health communication" OR "health promotion" OR "misinformation"))
Web of Science	TS=("memes" OR "internet meme" OR "viral content") AND TS=("health communication" OR "health promotion" OR "misinformation")

### Eligibility Criteria

To ensure methodological consistency and the relevance of the included studies, clear eligibility criteria were applied. This review considered for inclusion only peer-reviewed empirical research, encompassing qualitative, quantitative, or mixed-method designs, that explicitly addressed the use of memes or viral content within the scope of health communication. Studies were eligible if they were published between March 2020 and March 2025 and available in either English or Spanish. Priority was given to research that examined the role of these digital formats—such as image macros, short videos, GIFs, and remixable visual content commonly disseminated on platforms such as Instagram, TikTok, Twitter/X, and Facebook—in disseminating medical information, shaping public perception, mobilizing health professionals, engaging audiences, or addressing health-related misinformation. Studies were excluded if they were not peer-reviewed, lacked original empirical data (such as opinion pieces, editorials, or commentaries), or focused exclusively on general social media usage without specific reference to memes or viral content in health contexts. Publications released prior to 2020 or written in languages other than English or Spanish were also excluded from the review. Moreover, in alignment with the objectives of infoveillance [12], the selection process was informed by the need to monitor and interpret emerging patterns of health communication within digital meme ecosystems, acknowledging their potential as markers of infodemic activity. The detailed selection process is illustrated in the PRISMA 2020 flow diagram in the Results section.

### Selection Process

Duplicates were removed prior to screening. The selection process was conducted in 2 phases. In the first phase, titles and abstracts were independently screened by 3 reviewers (ACP, BJ-G, and CS-L) to assess preliminary relevance. In the second phase, full texts of the remaining papers were evaluated in detail, applying the predefined eligibility criteria to determine final inclusion. Discrepancies in paper selection were resolved through discussion and consensus among the 3 reviewers.

### Data Extraction and Thematic Organization

For each included study, relevant information was extracted, including authorship, publication year, study design, objectives, and key findings. The selected papers were then classified according to thematic domains aligned with the review’s analytical structure. These domains included the dual impact of social media in health communication, the influence of memes on public perceptions and behaviors, their role in mobilizing community engagement, issues surrounding digital literacy, the interaction between conspiracy theories and public health discourse, the emotional and cognitive mechanisms that underpin humor and misinformation, and finally, evidence-based strategies for combating misinformation, including prebunking, debunking, and combined intervention models.

While the review prioritizes primary empirical studies, relevant secondary literature (eg, systematic reviews and narrative syntheses) was also considered to support theoretical framing in the “Introduction” and “Discussion” sections but not included in the core analysis.

### Rigor and Limitations

Although this is a narrative review and not a systematic one, methodological transparency was ensured through clear articulation of the search strategy, criteria for inclusion and exclusion, independent review by 3 researchers, and structured thematic synthesis. Limitations inherent to narrative approaches—such as potential selection bias, the absence of formal quality appraisal, and the interpretative nature of synthesis—are acknowledged and addressed in the “Limitations and Future Research” section.

## Results

### Search Results

A total of 386 records were identified from 3 electronic databases: MEDLINE (120 records), Scopus (148 records), and Web of Science (118 records). After removing 17 duplicate records (4.4% of total), and excluding 268 records (69.4%) marked as ineligible by automation tools, 101 records remained for title and abstract screening.

**Figure 1.**
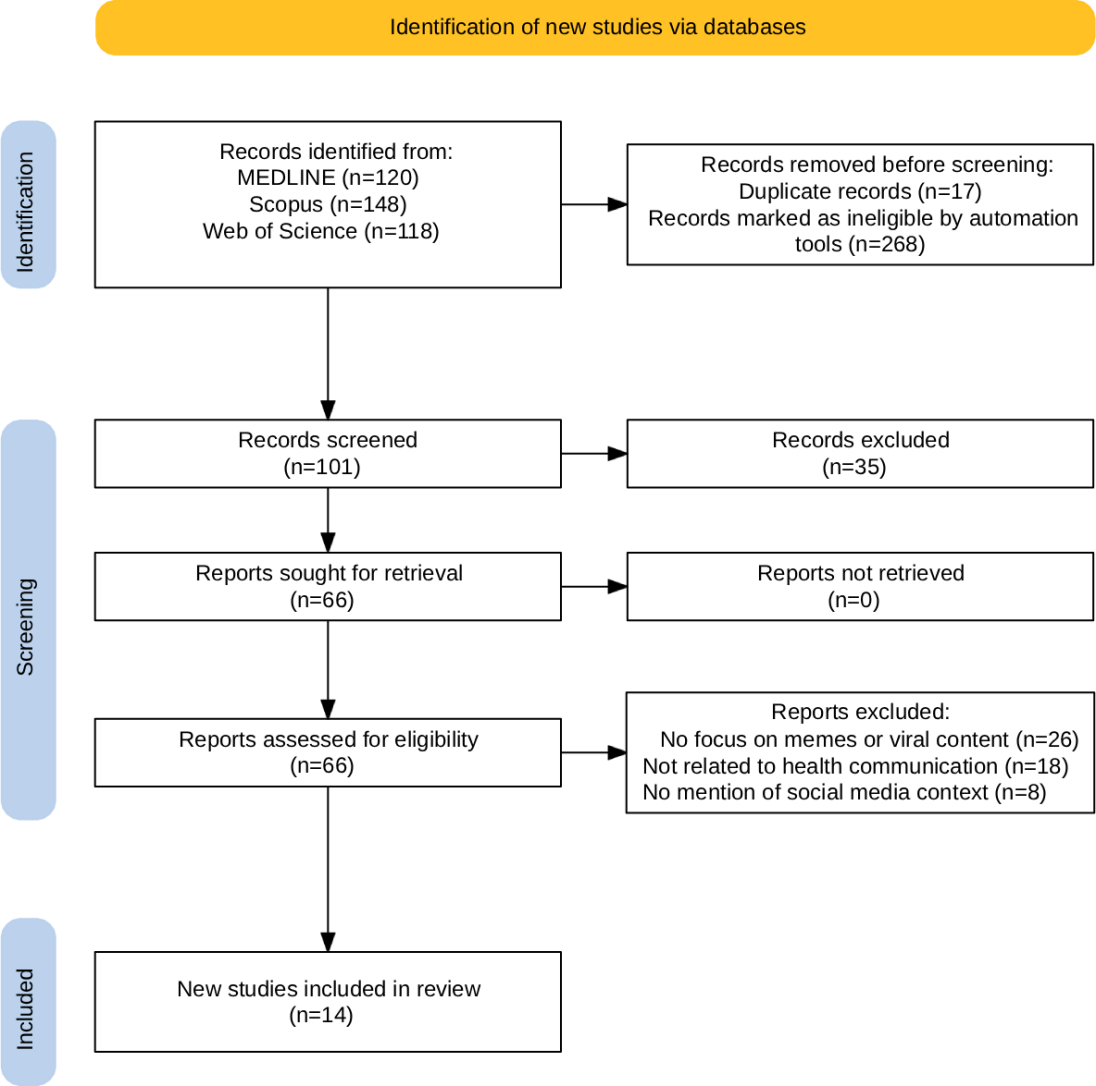
PRISMA (Preferred Reporting Items for Systematic Reviews and Meta-Analyses) diagram for selecting the publications from MEDLINE, Scopus, and Web of Science.

During the screening phase, 35 out of 101 records (34.7% of screened) were excluded for not meeting initial inclusion criteria. Of the 66 reports sought for retrieval, all were obtained (0% not retrieved). Upon full-text eligibility assessment, 52 out of 66 reports (78.8% of retrieved) were excluded for the following reasons: 26 out of 52 studies (50%) lacked focus on memes or viral content, 18 out of 52 studies (34.6%) were not related to health communication, and 8 out of 52 studies (15.4%) made no mention of social media context. Ultimately, 14 out of 66 studies (21.2% of assessed full texts) met all eligibility criteria and were included in this integrative review. The study selection process is detailed in the PRISMA flow diagram (Figure 1), illustrating the comprehensive filtering and screening steps undertaken to ensure methodological rigor and relevance.

### The Impact of Social Media on Health Perception and Misinformation

Social media memes have played a central role in shaping public responses to health crises. Martínez-Cardama and García-López [16] analyzed nearly 1000 Spanish memes shared during the early stages of the COVID-19 pandemic and found that their emotional tone evolved in tandem with societal sentiment—from denial and irony to acceptance and resilience. Far from being trivial, these visual micronarratives served as collective coping mechanisms, capturing the population’s shifting mood and transforming complex experiences into emotionally resonant formats that often proved more impactful than traditional public health messaging.

This dual potential of social media is echoed in Jalli’s [17] study on TikTok-based activism in Southeast Asia. Youth-led movements strategically used platform-native formats such as duets and stitched memes to mobilize civic action around health and social issues. However, the same affordances that fueled engagement also exposed activists to algorithmic volatility, coordinated harassment, and misinformation campaigns. The study highlights how viral content can simultaneously empower and endanger users, especially in politically unstable contexts.

Isik et al [18] identified a striking gap in how health information is perceived depending on users’ professional backgrounds. Their findings show that non–health care participants were more likely to accept health content on social media uncritically, often using it to inform medical decisions without consulting professionals. In contrast, health care professionals exhibited greater skepticism and reluctance to trust digital sources. This divergence underscores the risk of misinformation shaping public health behaviors, especially among audiences lacking specialized training.

In a broader review of digital misinformation, Altay et al [19] argue against alarmist narratives that exaggerate the reach of false content. They found that misinformation often originates from traditional media and is recycled online, where it competes with satire, critique, and social commentary. Memes, they suggest, are not inherently dangerous but must be understood within their cultural and rhetorical contexts—where humor and ideology often overlap. Their analysis calls for moving beyond simplistic metrics of exposure to embrace more nuanced interpretations of user intent and meaning-making.

Research also reveals that online misinformation affects trust in health care professionals. According to Forgie et al [20], exposure to sensational or misleading health content online may lead patients to question medical advice or turn to unproven alternatives, straining the doctor-patient relationship. Their findings highlight the urgent need for stronger digital literacy frameworks and more robust health communication strategies that anticipate and address the emotional drivers of misinformation.

Mheidly and Fares [21] respond to these challenges with a structured model for infodemic management. Their framework emphasizes timely, transparent, and empathetic communication during health crises, including proactive partnerships between medical institutions and digital platforms. Notably, they advocate for participatory messaging, where audiences are not just recipients but cocreators of health narratives—an approach especially pertinent in the fast-moving, emotionally charged environment of social media [21].

In the context of the Global South, Kubheka et al [22] examined how digital health promotion unfolds in South Africa. While social media platforms offer cost-effective channels for outreach, their potential is unevenly distributed. Structural barriers such as the digital divide, language fragmentation, and limited health literacy reduce impact and risk exacerbating inequalities. The authors call for a more inclusive, equity-oriented strategy—one that integrates cultural sensitivity and community participation into digital health efforts [22].

Finally, Bonnet and Sellers [23] caution that the very characteristics that make memes effective—brevity, humor, and visual impact—also make them potent carriers of misinformation. Their work underscores the need for public health interventions that harness digital virality while maintaining accuracy and promoting critical engagement.

Public perception of health risks is deeply influenced by the type and frequency of media consumed. Li and Zhong [24] found that individuals who rely heavily on digital media are more prone to experiencing heightened anxiety and fear during health crises. This increased emotional reactivity not only shaped behaviors such as mask wearing and social distancing but also led to exaggerated perceptions of threat, including panic buying and avoidance of essential health care.

These dynamics are further explained by media dependency theory, which suggests that during uncertainty, people gravitate toward sources they consider reliable [25]. While traditional media—especially television—had a stabilizing effect on emotions and fostered a sense of control, digital platforms tended to amplify fear and urgency. This aligns with Wakefield et al [26], who previously showed that conventional media remains a cornerstone of authoritative health communication, even in digital-first environments. The challenge for public health communicators is to balance emotional impact with informational accuracy. While fear-based messaging can prompt immediate compliance, it risks eroding trust over time. More effective strategies emphasize self-efficacy, reinforce positive behaviors, and draw on credible, culturally resonant sources.

Memes occupy a unique position in this emotional economy. Basch et al [27] showed that memes promoting health behaviors such as mask wearing gained significant traction during the pandemic. However, many others mocked public health measures or spread conspiracy theories, revealing the double-edged role of humor. A systematic review by Gabarron et al [28] confirmed that memes were frequently used to disseminate false claims about COVID-19 treatments and preventive measures, illustrating how easily engaging formats can obscure misinformation behind jokes or satire.

In contrast to the harmful potential of humor-driven content, Griffith et al [29] explored how memes can also function as tools for resilience and community cohesion during public health crises. Their qualitative study analyzed the role of meme sharing among sexual minority men and nonbinary individuals during the COVID-19 pandemic, revealing how humor served not merely as a distraction but as a form of emotional regulation and collective coping. Memes provided users with a way to process uncertainty, reinforce identity, and maintain social connection under conditions of stress and isolation. This study highlights that meme engagement, particularly when occurring within affirming digital spaces, can offer psychosocial benefits that go beyond entertainment, functioning as informal mechanisms of mental health support. These findings emphasize the duality of meme cultures: while they can amplify misinformation, they also foster resilience and solidarity, particularly among marginalized communities navigating health-related adversity.

### Conspiracy Theories and Public Health Memes

Memes have become powerful carriers of conspiracy theories during public health crises, merging visual wit with emotionally provocative messaging. Buts [30] illustrates this with antivaccination memes featuring Mahatma Gandhi and mercury references, which blend historical authority with imagery designed to elicit moral disgust. These memes operate through ambiguity, often subverting their own surface meaning and making it harder to identify and debunk falsehoods. Their rhetorical power lies in leveraging irony and familiarity to bypass rational scrutiny, reinforcing distrust while masking ideology under humor.

Farhart et al [31] deepen this analysis through a large-scale survey, identifying 3 key motivations behind conspiracy meme sharing: genuine belief, alarm signaling to one’s in-group, and a disruptive “need for chaos.” While belief remains the strongest predictor, the latter motive reflects a broader desire to destabilize rather than persuade. For some users, spreading misinformation is performative defiance—not misinformation as persuasion but as provocation.

This interplay between identity, emotion, and visual rhetoric intensifies during health emergencies. As van Prooijen and Douglas [32] argue, moments of uncertainty fuel conspiracy beliefs by offering agent-based explanations that feel more intuitive than scientific complexity. This tendency was visible during the COVID-19 pandemic, where narratives about virus origins, treatments, and mandates spread rapidly via memes. Wang et al [33] identified 3 mechanisms behind this dynamic: algorithmic amplification of engaging content, echo chambers that reinforce existing beliefs, and the appeal of cognitive simplicity. These factors not only accelerate misinformation but insulate it from correction.

The role of memes in this environment is particularly insidious because of their emotional stickiness. Panchal and Jack [34], studying forensic patients with a psychiatric disorder, observed how memes fostered an “us versus them” mentality, casting health professionals as oppressive figures rather than trusted advisors. Similarly, Pascual-Ferrá et al [35], analyzing 26,736 Facebook posts during the avian influenza outbreak, found that memes quickly framed public understanding—often mixing scientific facts with ideological tropes such as food regulation skepticism or antigovernment sentiment. Their study underscores the potential of meme monitoring as an early-warning tool for health agencies to detect misinformation trends before they entrench.

The broader problem, as shown in large-scale diffusion studies by Vosoughi et al [36], is that falsehoods—especially emotionally charged ones—spread faster and farther than verified information. Contrary to popular belief, this is not primarily driven by bots but by people. False health claims often provoke stronger emotional reactions, increasing their likelihood of being shared. Memes, by design, condense these claims into viral-ready formats—brief, humorous, and emotionally loaded—making them ideal misinformation vehicles.

Attempts to counteract this trend face significant hurdles. Ecker et al [37] describe the “continued influence effect,” in which misinformation persists even after correction. This has shifted the focus toward prebunking—preemptively exposing users to misleading tactics. Studies suggest that prebunking is more effective than reactive fact-checking, particularly when delivered in emotionally engaging, culturally attuned formats.

### Humor, Misinformation, and Emotional Engagement

Memes blend humor, visual wit, and ideological commentary, making them powerful tools for emotional engagement in health communication. Their brevity and multimodal nature allow them to not only simplify complex information but also render them susceptible to distortion. Arailopoulos et al [38] highlight how the adaptability of meme formats—image macros, object labels, and screenshots—drives their virality, enabling them to act as cultural signifiers and emotional amplifiers.

Mihăilescu [39] adds that meme creators are often intentional actors and not passive entertainers. They view their work as a means to shape narratives, challenge dominant discourses, and mobilize public sentiment. Their humor is strategic—using irony and parody to spark engagement while maintaining plausible deniability. This blurring between satire and misinformation complicates efforts to hold digital communicators accountable, particularly during health crises.

Anderau and Barbarrusa [40] categorize memes as low-reputation, high-impact communicative tools. They operate through “context collapse,” allowing disparate references and emotions to converge in a single visual frame. While this enhances resonance and shareability, it also risks oversimplification and ideological reinforcement. Their taxonomy identifies humor, identity signaling, and emotional valence as core traits in the spread of misinformation.

Importantly, memes are not exclusively harmful. Marx et al [41] show how Brazilian public health agencies used Twitter memes to combat vaccine hesitancy. By incorporating pop culture, local humor, and meme-native framing, these campaigns increased public engagement and countered disinformation narratives. Similarly, Skórka et al [42] found that meme search spikes aligned with COVID-19 mortality peaks, suggesting that people used humor as a coping mechanism. Their sentiment analysis revealed a mix of positive and negative tones—memes simultaneously validated collective fears and diffused them through laughter. Memes can normalize protective behaviors such as mask wearing by framing them as socially shared experiences. However, this potential must be carefully managed. Without grounding in accurate information, humor risks trivializing public health efforts or, worse, endorsing harmful behaviors.

Yet, the emotional power of memes also fuels polarization. Zollo et al [43] demonstrated that conversations around conspiracy content grow increasingly negative over time, a trend that also affects discussions about scientific topics. This escalation intensifies division and reduces receptivity to corrective messages. Farhoudinia et al [44] support this with machine learning models showing that fake news—especially when meme-based—triggers strong emotions such as anger, disgust, and fear. These emotional responses not only increase virality but also reduce critical engagement.

The challenge, then, lies in distinguishing between memes that foster resilience and those that reinforce misinformation. Díaz Ruiz and Nilsson [45] caution that in identity-driven echo chambers, memes are judged less by accuracy than by emotional and ideological fit. Users gravitate toward familiar narratives—even if false—because they offer social cohesion and expressive power. In such environments, truth becomes secondary to affirmation.

### Mitigating Health Misinformation

Combating health misinformation requires a proactive and multilayered strategy—one that combines emotional intelligence with structural interventions [46]. Van der Meer and Jin [47] showed that detailed factual corrections are more effective than brief rebuttals, especially when issued by trusted institutional sources. This highlights the need for credible voices and emotionally sensitive framing in health messaging, particularly during crises.

Building on this, Eysenbach [13] proposed a comprehensive infodemic management framework built on 4 pillars: information monitoring, digital literacy, knowledge refinement, and accurate knowledge translation. These pillars underscore that combating misinformation is not just a matter of correcting facts but of building systemic resilience. Misinformation, after all, spreads not only due to cognitive failure but also due to emotional and social dynamics—fear, distrust, and group identity.

A critical distinction in misinformation control is the difference between *prebunking* (inoculation before exposure) and *debunking* (correction after exposure). Roozenbeek and van der Linden [48] demonstrated that prebunking enhances users’ ability to recognize and resist misinformation, especially when it is delivered in engaging, gamified formats. Debunking, in contrast, often struggles against the *continued influence effect* [37], where misinformation lingers in memory despite correction.

Recent experimental studies reinforce this view. Barman and Conlan [49] found that prebunking significantly improved participants’ accuracy in identifying misinformation, although effectiveness varied across political orientations. Tay et al [50] showed that meme-based prebunking—presenting common misinformation tactics through humor and visual cues—was particularly successful in reducing the spread of implied or emotionally charged falsehoods.

However, misinformation is not easily undone. Ecker et al [37] emphasize that without a clear alternative explanation, corrections often fail. Nyhan and Reifler [51] go further, identifying the *backfire effect*, where factual corrections may reinforce preexisting false beliefs, especially when those beliefs align with political or identity-based convictions. These findings underscore that purely informational strategies are insufficient without emotional and cognitive alignment.

To counteract this, Roozenbeek and van der Linden [52] developed “The Fake News Game,” a media literacy intervention rooted in inoculation theory. Participants who engaged in misinformation creation exercises developed greater resistance to false content, suggesting that participatory models are more effective than passive exposure to corrections.

Effectiveness improves further when these strategies are integrated. Bragazzi and Garbarino [53] argue that misinformation persists not because it is persuasive but because it satisfies psychological and social needs—simplifying uncertainty, affirming group identity, and offering emotional closure. Thus, interventions must address both the *content* and the *function* of misinformation.

Silesky et al [54] demonstrated the value of this approach in a study targeting COVID-19 misinformation in Hispanic communities. Their success relied on blending social media monitoring, influencer collaboration, and culturally tailored educational messaging. By acting preemptively and through trusted networks, the campaign improved information retention and reduced susceptibility to misinformation.

Another strategy is source discreditation—undermining the perceived credibility of misinformation sources. Ecker et al [55] found that exposing conflicts of interest or historical falsehoods diminished public trust in misinformation sources, especially when paired with factual corrections. Combining source discreditation with narrative replacement helps audiences both reject falsehoods and adopt accurate alternatives.

Cognitive and behavioral training also enhances misinformation resistance. Ishizumi et al [56] report that interventions focused on critical thinking and peer education build long-term resilience. Empowering individuals to become *correctors* within their communities strengthens their engagement and deepens their understanding, creating social ripple effects.

## Discussion

### Principal Findings

The findings of this review reveal that internet memes occupy a paradoxical space in health communication: they are simultaneously tools of simplification and distortion. Their visual, humorous, and emotionally resonant nature makes them highly effective for engaging audiences and translating complex medical information into relatable formats. As reported in multiple studies, particularly during the COVID-19 pandemic, memes played a significant role in normalizing preventive behaviors such as vaccination or mask wearing by embedding them in culturally familiar, emotionally appealing content [5,57].

However, these same characteristics make memes vulnerable to misuse. Their brevity and viral design often favor emotional appeal over informational accuracy, leading to oversimplification or misinterpretation. Conspiracy-driven and chaos-driven memes have exploited these dynamics to erode trust in public health institutions, particularly in politicized or polarizing contexts [31,58]. This dual capacity—empowering and destabilizing—positions memes as a double-edged instrument in the digital public health landscape. In addition, to optimize health communication, it is essential to critically evaluate intervention strategies such as prebunking and digital literacy initiatives. Future research should address how these measures can be prioritized, determine which approach may yield the greatest effectiveness, and assess the practical viability of their real-world application. Such an analysis will be pivotal in guiding health institutions toward interventions that both engage audiences and robustly counteract misinformation.

### Implications for Public Health Communication

Crucially, the success of health communication via memes depends not only on their esthetic appeal but also on the credibility and authenticity of their sources. Jenkins et al [59] found that users rely on heuristics such as tone, expertise cues, and community endorsement (likes and shares) to judge the reliability of health messages on platforms such as Twitter and Facebook. However, as Helou et al [60] highlight, the frequent lack of conflict-of-interest disclosures among influencers undermines perceived trustworthiness, especially when health content is monetized or promotional in nature.

This affects not only individual perception but also collective behavior. As several reviews indicate, memes can amplify public understanding of health recommendations when integrated into well-designed campaigns [8,61], but they also reinforce misinformation when emotionally charged content spreads without verification. Farrokhi et al [62] demonstrated that culturally adapted, meme-based messages improved oral health engagement among underserved communities—underscoring their potential when thoughtfully deployed. While humor and memes can enhance engagement and emotional connection with audiences, it is critical to draw ethical boundaries to prevent the trivialization of sensitive health content. To avoid mirroring the same emotional tactics exploited by misinformation, we must strike a careful balance between reach and responsibility—ensuring that clarity, accuracy, and respect for the patient experience remain central to any message we share.

At the community level, memes can function as tools of digital advocacy. When aligned with shared values and experiences, they foster participation, identity, and mobilization—particularly in peer-to-peer spaces where traditional health messaging often fails to penetrate. Yet, their decentralized and remixable nature complicates efforts to ensure message consistency or factual integrity. Brzozowska and Gotlib [63] argue that memes in skin health promotion gained traction precisely because of their emotional relevance and esthetic accessibility—features rarely emphasized in official communications.

In response to the growing threat of memetic misinformation, researchers have explored both prebunking and debunking strategies. Prebunking, grounded in inoculation theory, shows promise in building resistance to falsehoods before exposure—especially when interactive or gamified [58]. Yet, the reactive nature of debunking remains dominant, despite its limited efficacy in meme-based environments. As Henderson and Gow [64] note, the most impactful interventions are those that embed corrective content within culturally and visually familiar formats, rather than relying solely on textual rebuttals.

These findings underscore the urgency of integrating infodemiological frameworks into public health communication. As the memetic dimension of health narratives becomes increasingly relevant, public health actors must develop strategies that anticipate and respond to digital infodemics. Eysenbach’s infodemiology model [13] provides a critical lens through which to understand memes not only as cultural artifacts but also as informational agents capable of amplifying or mitigating health risks, lately supported by Schüz and Jones [65]. Recommendations for practitioners include leveraging meme trends for early infoveillance, pretesting memetic content in diverse populations, and designing proactive prebunking campaigns that align with platform-specific engagement patterns. From an infodemiological perspective, the strategic monitoring of viral content—particularly memes—offers valuable opportunities for early detection of misinformation outbreaks. Health institutions should incorporate real-time memetic trend analysis into their infoveillance systems and collaborate with digital content creators to coproduce culturally resonant, scientifically grounded messages. Embedding meme-based strategies within broader infodemic management plans can enhance public trust, reduce information fatigue, and improve the reach and retention of health messages during future crises. Future research should explicitly prioritize these measures, evaluate which interventions are most effective, and examine the practical feasibility of their application in diverse real-world health communication contexts.

Ultimately, navigating the memetic terrain requires more than technical skill or fact-checking infrastructure. It demands a deeper cultural literacy and a willingness to collaborate with digital communities, influencers, and content creators. Memes must not be viewed as marginal or frivolous but as contemporary vehicles of meaning-making—capable of both advancing and undermining public health goals. To engage effectively in this space, public health institutions must evolve, embracing humor without compromising accuracy, leveraging emotion without manipulation, and integrating digital fluency into the core of health communication strategy.

### Limitations and Future Research

While this review highlights a growing body of empirical work on the role of memes in health communication, several notable gaps persist in the literature—both in terms of scope and methodological approach. First, a substantial proportion of the existing research remains focused on the COVID-19 pandemic, which, while contextually rich, may limit the generalizability of findings to other public health challenges. Memes related to chronic illness, mental health, reproductive health, environmental health, and health equity remain significantly underexplored, despite the relevance of these topics in online discourse.

Second, most studies identified rely on descriptive or cross-sectional designs, focusing on content analysis or short-term audience reactions. While valuable for mapping trends and themes, these approaches often fall short of capturing the long-term behavioral or attitudinal impacts of health-related memes. There is a pressing need for longitudinal and experimental studies that assess how memes influence decision-making, health behaviors, and trust in public institutions over time.

Moreover, very few studies address the role of meme creators themselves—those individuals or collectives who produce and shape the digital rhetoric circulating in health contexts. Understanding their motivations, creative strategies, and ethical considerations could provide key insights into how public health actors might collaborate with or learn from these informal communicators.

There is also limited exploration of how platform-specific dynamics—such as algorithmic amplification, moderation policies, and visual affordances—influence the spread and reception of health-related memes. These digital ecologies are not neutral backdrops but active agents that shape who sees what, when, and how. As such, future research would benefit from an interdisciplinary approach that integrates public health, media studies, data science, and digital anthropology.

There is a critical need to include diverse cultural and linguistic contexts in the study of health memes. Much of the literature remains Anglocentric, overlooking how meme culture operates across different regions, populations, and health systems. Comparative and multilingual studies could reveal distinct patterns of engagement, resistance, or adaptation that enrich our understanding of memes as global health communication tools. Addressing these gaps will require methodological innovation, interdisciplinary collaboration, and a willingness to take internet culture seriously—not as a trivial domain but as a dynamic space where health knowledge is negotiated, contested, and reimagined.

### Conclusions

This review sheds light on the complex and often contradictory role that internet memes play in health communication. Far from being mere online distractions, memes have become powerful tools for shaping how people engage with and understand health information. Their visual impact, humor, and emotional tone make them especially effective in simplifying complex messages and reaching a wide range of audiences—particularly those who might not respond to traditional forms of public health messaging. Although the current body of evidence highlights the potential of memes as tools for health communication—particularly in fast-paced digital environments—this strategy should be viewed as a promising but still evolving approach. Given the predominance of COVID-19–related studies, the lack of longitudinal data, and a language bias toward English language content, further research is needed to evaluate both the short- and long-term impacts of meme-based messaging across diverse populations and health topics. Memes may offer a valuable addition to public health communication efforts, especially in contexts where engagement and emotional resonance are key, but their use should be guided by ongoing critical evaluation and contextual sensitivity.

Yet, these same qualities can also work against public health goals. The very features that make memes engaging—brevity, irony, and virality—can lead to oversimplification, misinterpretation, or even the spread of misinformation. As the evidence shows, memes can reinforce helpful health behaviors, but they can also amplify doubt, fuel conspiracy theories, or undermine trust in health authorities, depending on how they are framed and who shares them. Moreover, memes have demonstrated potential in enhancing the training and awareness of health care professionals, offering innovative avenues for continuous education and professional mobilization.

These findings suggest that public health professionals and institutions need to move beyond conventional, top-down communication strategies. Instead, they should embrace a more participatory, culturally aware, and digitally savvy approach—one that sees memes not just as potential threats but as opportunities to connect, inform, and empower. This includes working collaboratively with creators and online communities, crafting content that is both engaging and evidence-based, and investing in media literacy to help people navigate the fast-paced, emotionally charged world of digital health content.

In short, memes matter. They are not a passing trend but a central feature of how health narratives are constructed, contested, and circulated online. If used wisely, they can become a powerful ally in promoting accurate information and public trust. But to do so, we must meet audiences where they are—not only with science but also with creativity, empathy, and a deep understanding of the digital cultures they inhabit.

## Supplementary material

10.2196/77029 Checklist 1 PRISMA checklist.

## Abbreviations

MeSH: Medical Subject Headings
PHI: public health influencer
PRISMA: Preferred Reporting Items for Systematic Reviews and Meta-Analyses
