# Supplementary material for: Internet Memes as Drivers of Health Narratives and Infodemics: Integrative Review
Source: JMIR Infodemiology. 2025 Dec 19;5:e77029. doi: 10.2196/77029 (PMC12716833; doi:10.2196/77029)
Supplement: Checklist 1 [file infodemiology-v5-e77029-s001.docx]

# PRISMA 2020 checklist—integrative review

Internet Memes as Drivers of Health Narratives and Infodemics: An Integrative Review

Carmona Pestaña et al., 2025

| Section | Item | Checklist Item | Location in Manuscript |
| --- | --- | --- | --- |
| TITLE | 1 | Identify the report as a systematic review. | Not applicable – integrative review; title identifies type. |
| ABSTRACT | 2 | Structured summary. | Abstract (page 1) |
| INTRODUCTION | 3 | Rationale. | Introduction (pp.1–3) |
| INTRODUCTION | 4 | Objectives. | Objectives section (p.3) |
| METHODS | 5 | Eligibility criteria. | Eligibility Criteria (p.4) |
| METHODS | 6 | Information sources. | Search Strategy (p.3) |
| METHODS | 7 | Search strategy. | Search equations detailed (p.3) |
| METHODS | 8 | Selection process. | Selection Process + PRISMA Diagram (p.4) |
| METHODS | 9 | Data collection process. | Data Extraction (p.5) |
| METHODS | 10 | Data items. | Data Extraction (p.5) |
| METHODS | 11 | Risk of bias assessment. | Not applicable – no formal tool; noted in Limitations |
| METHODS | 12 | Effect measures. | Not applicable – narrative synthesis |
| METHODS | 13 | Synthesis methods. | Narrative thematic synthesis (pp.5–8) |
| RESULTS | 14 | Study selection. | Search Results + PRISMA (p.5) |
| RESULTS | 15 | Study characteristics. | Results thematic sections (pp.5–8) |
| RESULTS | 16 | Risk of bias. | Not applicable – no RoB assessment |
| RESULTS | 17 | Results of individual studies. | Summaries throughout Results |
| RESULTS | 18 | Synthesis results. | Thematic synthesis across Results |
| DISCUSSION | 19 | Summary of evidence. | Principal Findings (pp.8–9) |
| DISCUSSION | 20 | Limitations. | Limitations and Future Research (pp.9–10) |
| DISCUSSION | 21 | Conclusions. | Conclusions (p.10) |
| OTHER | 22 | Registration / protocol. | No protocol (narrative review) |
| OTHER | 23 | Support. | Funding section (p.10) |
| OTHER | 24 | Competing interests. | Conflicts of Interest (p.10) |
| OTHER | 25 | Availability of data. | Not applicable – no dataset generated |
